# Biomechanics of subcellular structures by non-invasive Brillouin microscopy

**DOI:** 10.1038/srep37217

**Published:** 2016-11-15

**Authors:** Giuseppe Antonacci, Sietse Braakman

**Affiliations:** 1 Department of Physics, Imperial College London, UK; 2 Centre for Life Nano Science@Sapienza, Istituto Italiano di Tecnologia, Rome, Italy; 3 Department of Bioengineering, Imperial College London, UK

## Abstract

Cellular biomechanics play a pivotal role in the pathophysiology of several diseases. Unfortunately, current methods to measure biomechanical properties are invasive and mostly limited to the surface of a cell. As a result, the mechanical behaviour of subcellular structures and organelles remains poorly characterised. Here, we show three-dimensional biomechanical images of single cells obtained with non-invasive, non-destructive Brillouin microscopy with an unprecedented spatial resolution. Our results quantify the longitudinal elastic modulus of subcellular structures. In particular, we found the nucleoli to be stiffer than both the nuclear envelope (p < 0.0001) and the surrounding cytoplasm (p < 0.0001). Moreover, we demonstrate the mechanical response of cells to *Latrunculin-A*, a drug that reduces cell stiffness by preventing cytoskeletal assembly. Our technique can therefore generate valuable insights into cellular biomechanics and its role in pathophysiology.

The biomechanical behaviour of cells is implied to be extensively involved in the development of diseases such as atherosclerosis, cancer and glaucoma. In these diseases, changes in cellular stiffness and/or contractility coincide with the onset of the disease. In atherosclerosis, disturbed and oscillatory wall shear stress coincide with plaque formation at arterial branch points through a process that is mediated by the shear-sensitive endothelial cells that line the artery^1^. In glaucoma, increased endothelial stiffness is associated with increased outflow resistance and intraocular pressure, which can lead to blindness^2^. In breast cancer, malignant human breast epithelial cells exhibited a significantly reduced apparent stiffness compared to their non-cancerous counterparts, potentially aiding cell migration in metastasis^3^. However, our understanding of how molecular and subcellular components contribute to cell stiffness and how cellular mechanical properties contribute to the pathogenesis of these diseases remains limited. A new field of nano-biomechanics is now emerging to better understand subcellular mechanics^4^.

One factor that currently limits progress in the field of nano-biomechanics is the methods available to measure the mechanical properties of cells. Atomic force microscopy (AFM) is currently the gold standard with a resolution in biologic samples down to nanometer scales^5^,^6^. However, AFM is invasive because it involves nanoindentation, which could invoke a cellular reaction. Moreover, AFM cannot probe inside the cell because it is limited to surface topologies of cells cultured on 2D flat substrates. Other techniques, such as optical tweezers^7^, deformability cytometry^8^, and micropipette aspiration^9^, measure cellular deformation by applying shear stresses or pressure gradients in suspension, which renders these techniques both invasive and subject to strong non-linear effects^10^. By contrast, standard non-invasive elastography techniques, such as ultrasound^11^ and magnetic resonance imaging^12^, are inherently limited by a low spatial resolution.

To overcome these limitations, we present Brillouin microscopy as a method to non-invasively and non-destructively assess intracellular stiffness with a submicron resolution. Confocal Brillouin microscopy uses light to yield three-dimensional mechanical images by probing local spontaneous acoustic waves propagating along the sample^13^, therefore with no contact and label requirements^14^. The high-frequency longitudinal elastic modulus (M′) of a viscoelastic material is obtained by measuring the frequency shift of the light scattered inelastically by local spontaneous acoustic waves (see Methods)^15^,^16^. Up to now, Brillouin microscopy has been used to characterise the biomechanical properties of the lens cornea^17^; to quantify plaque stiffness in atherosclerosis^18^; to screen bacterial meningitis^19^; and to assess cytoskeleton stiffening^20^. Spectral broadening arising from the collection of photons by high numerical aperture (NA) lenses had limited the technique to a low spatial resolution^13^,^21^. Nevertheless, a recent study has shown that the broadening is minimised in the backscattering geometry, therefore suggesting the potential use of high NA (>0.5) optics^22^. In this work, we obtained an unprecedented submicron spatial resolution to enable the mechanical characterisation of individual subcellular structures using a custom-built confocal Brillouin microscope.

## Results

### Validation of confocal Brillouin microscopy

The gigahertz frequency domain associated with the spontaneous acoustic phonons results in a higher elastic modulus than the Young’s modulus (E) obtained by standard quasi-static methods that use, for example, AFM and rheometer configurations. While the longitudinal elastic modulus measures the ratio of an applied uniaxial stress to the material strain assuming a confined displacement along the longitudinal direction, the Young’s modulus measures the stress-strain ratio by taking into account displacements also in the other directions. Moreover, the compressibility of materials is differently considered by the two moduli. As a result, the longitudinal modulus and the Young’s elastic modulus are different and cannot be easily related.

**Figure 1:**
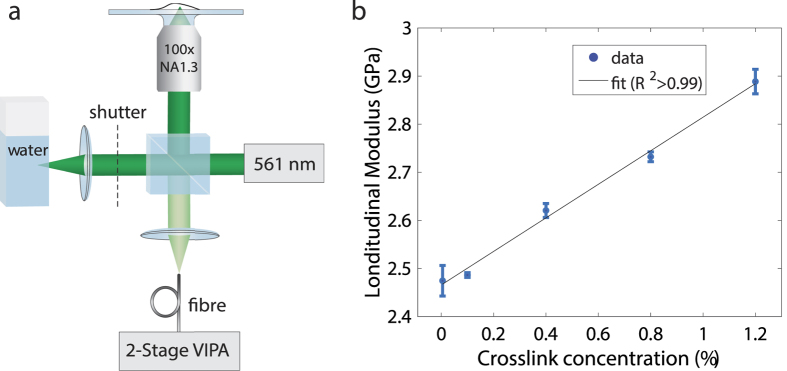
Confocal Brillouin microscope and stiffness calibration. Optical setup diagram of the confocal Brillouin microscope (**a**) and longitudinal modulus of polyacrylamide hydrogels of different stiffness (**b**). The stiffness of the polyacrylamide gels was varied by adjusting the amount of crosslinker from 0.05% to 1.2% w/v bisacrylamide. The hydrogels (n = 3 for each crosslink concentration) were measured using the Brillouin microscope to determine their longitudinal modulus. The longitudinal modulus appears to increase linearly with the hydrogel crosslink concentration (R^2^ > 0.99), indicating that Brillouin light scattering provides a meaningful measure of bulk stiffness.

To show that changes in the longitudinal modulus are indicative of stiffness variations, we measured polyacrylamide hydrogels of different crosslink concentrations using the custom-built confocal Brillouin microscope (Fig. 1a), which provided a subcellular spatial resolution of ~0.3 × 0.3 × 0.7 μm^3^ (see Methods). Figure 1b shows a linear increase in the longitudinal modulus as a function of the crosslink concentration, with the latter being directly associated with the stiffness of polyacrylamide hydrogels^23^. It needs to be noticed that for non-amorphous materials, such as cells and biological tissues, the elasticity tensor is not diagonal and the c_11_ stiffness tensor element measured in Brillouin light-scattering microscopy cannot be simply associated with the bulk modulus^24^. As a result, the Brillouin measurements reported in this manuscript should be thought as a mean longitudinal modulus, which takes into account the other acousto-optic tensor elements defined by the symmetry of the material investigated. Although both the refractive index and the density can be non-uniform across biological tissues and organisms, recent results have demonstrated that the longitudinal modulus is not substantially affected by these changes^20^,^25^. These findings endorse Brillouin microscopy as a reliable technique to assess cellular stiffness.

### Subcellular biomechanical imaging

**Figure 2:**
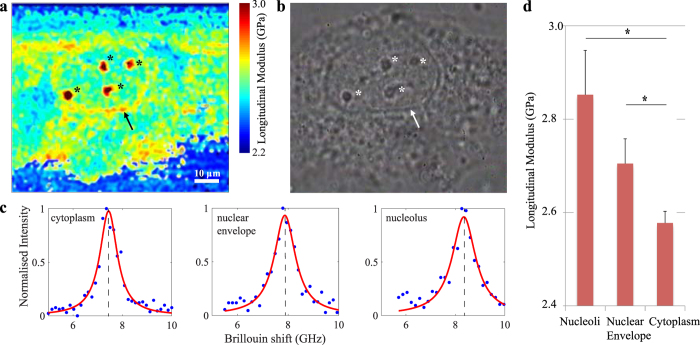
High-resolution Brillouin image of a single cell *in vitro.* A cross section through a single cultured human umbilical vein endothelial cell imaged using both Brillouin (**a**) and phase contrast (**b**) microscopy at 100x magnification. Cellular structures, such as the nuclear envelope (arrow) and nucleoli (*), are clearly recognisable in both image modalities. Representative Brillouin (Anti-Stokes) spectral peaks from cytoplasm, nuclear envelope and nucleoli (**c**). From the Brillouin shift and the associated longitudinal modulus, it is apparent that these structures exhibit different mechanical properties compared to the surrounding cytoplasm. In particular, the nuclear envelope and nucleoli exhibit higher longitudinal moduli (2.78 ± 0.05 GPa, p < 0.0001 and 3.12 ± 0.07 GPa, p < 0.0001 respectively) than the nucleus and cytoplasm that surrounds them (2.51 ± 0.04 GPa) (values reported as mean ± s.d.). A bar-plot represents the differences (mean ± SEM) between the longitudinal modulus as measured in the cytoplasm, nucleoli, and nuclear envelope of all experiments (*p < 0.001) (**d**).

The submicron resolution obtained with this confocal Brillouin microscope enabled the biomechanical characterisation of distinctive subcellular structures. The two panels in Fig. 2 show a Brillouin image and a phase contrast image of a single porcine aortic endothelial cell (PAEC). In both image modalities the outline of the nuclear envelope and four nucleoli are clearly visible. Given their separate Brillouin frequency shifts (Fig. 2c), these structures exhibit a significantly different longitudinal modulus to indicate their distinct biomechanical properties. In particular, the nucleoli present in the cell’s nucleus exhibited a longitudinal modulus of 2.85 ± 0.09 GPa and are stiffer compared to the surrounding cytoplasm, of which the longitudinal modulus was measured 2.58 ± 0.03 GPa (p < 0.0001, n = 4 experiments). All values are reported as mean ± standard error of the mean (SEM), unless stated otherwise. Similarly, where distinguishable in these cells, the nuclear envelope showed a higher longitudinal modulus of 2.71 ± 0.05 GPa compared to the cytoplasm (p < 0.0001, n = 2). The diagram in Fig. 2d shows the differences between the longitudinal modulus as measured in the cytoplasm, nucleoli, and nuclear envelope of all experiments. A comprehensive overview of the measurements of each experiment is included in Supplementary Figure S1.

### Cellular stiffness in response to external stimuli

**Figure 3:**
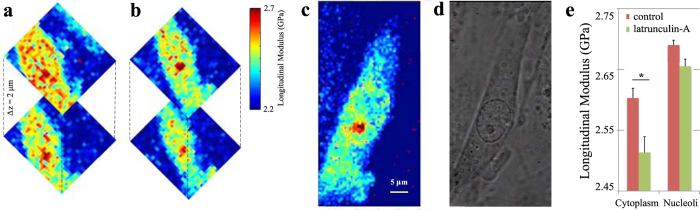
Cellular stiffness in response to latrunculin-A. Two cross sections along the z-axis through a cell before (**a**) and after (**b**) exposing the cell to latrunculin-A. A Brillouin image of the same cell taken at higher sampling resolution after drug exposure (**c**) and the associated phase-contrast image (**d**). Latrunculin is a toxin that prevents polymerisation of actin filaments and thereby decreases cell stiffness. Note that actin filaments are predominantly found in the cytoplasm and are absent from nucleoli. Indeed, cytoplasmic stiffness is decreased from 2.57 ± 0.05 to 2.46 ± 0.06 GPa (p < 0.0001), whereas nucleolar stiffness is not substantially affected by latrunculin-A exposure with a small decrease from 2.68 ± 0.07 GPa to 2.64 ± 0.06 GPa (p = 0.65) (values reported as mean ± s.d.). A bar-plot represents the change (mean ± SEM) in the longitudinal modulus of the cytoplasm and nucleoli in response to latrunculin-A of all experiments (*p < 0.001) **(e)**. These data show that Brillouin microscopy is capable of measuring both spatial and temporal variations in stiffness in a physiologically relevant stiffness range that facilitates the study of cellular biomechanics.

To show that Brillouin microscopy is not limited to surface topologies, we investigated changes in the longitudinal modulus in different z-sections of a single cell. Figure 3a shows a PAEC cell imaged at different depths to establish a baseline measurement of its subcellular structure. The mean longitudinal modulus of the cytoplasm was 2.60 ± 0.02 GPa, while the longitudinal modulus of the nucleolus was 2.69 ± 0.01 GPa. A second baseline measurement was taken at a later point in time, which showed that the longitudinal modulus did not change significantly over time (p = 0.3). Subsequently, latrunculin-A was added to the imaging medium at a final concentration of 500 nM. Latrunculin-A is a drug that lowers cell stiffness by preventing the formation of cytoskeletal actin filaments, which are omnipresent in the cytoplasm but which are mostly absent from nucleoli. The same cell was then imaged again with both Brillouin (Fig. 3b,c) and phase-contrast (Fig. 3d) image modalities to investigate the effect of latrunculin-A on the stiffness of the cell’s structures. To compare the Brillouin measurements before and after the addition of latrunculin-A, all measurements were normalised to the longitudinal modulus of the life cell imaging solution, which was measured to be 2.23 ± 0.03 GPa (p < 0.001). In response to latrunculin-A, cytoplasmic stiffness decreased by 3.6% to 2.51 ± 0.03 GPa (p < 0.0001, n = 3). This result is consistent with previous AFM measurements of cell stiffness in response to latrunculin-A^26^. Nucleoli were clearly distinguishable in the phase contrast image of two of these three cells, allowing to explore the effect of latrunculin-A on the mechanical properties of these structures. Interestingly, nucleolar stiffness appeared less affected by latrunculin-A as their stiffness decreased by 1.1% to 2.66 ± 0.01 GPa (p = 0.51). The diagram in Fig. 3e represents the change in the longitudinal modulus of the cytoplasm and nucleoli in response to latrunculin-A of all experiments. A comprehensive overview of the measurements of each experiment is included in Supplementary Figure S2.

## Discussion

The results presented here show that Brillouin microscopy can be successfully used to study the subcellular biomechanical properties of cells. The technique was used to mechanically characterise a three dimensional volume of cells along both transverse and axial axes, demonstrating that the technique is not limited to investigating surface topologies. The confocal Brillouin microscope was also used to investigate changes in the mechanical properties of cells in response to a chemical stimulus in the form of the cytoskeletal drug latrunculin-A, demonstrating the versatility and applicability of Brillouin microscopy to further the field of cellular biomechanics.

More established experimental methods to investigate cellular biomechanics, such as atomic force microscopy (AFM), microrheology^27^ or nuclear tracking during cell motility using microfluidic devices^28^, have mostly been indirect and invasive, which has limited the mechanical characterisation to surface topologies of cells. Uniquely, the non-invasive nature of Brillouin microscopy removes mechanical contact as a confounding factor associated with aforementioned invasive mechanical characterisation techniques. Removing this confounding factor is especially important when studying the effect of mechanical stimuli on cells. Using an oil-immersion objective lens of high numerical aperture (NA = 1.3) in a backscattering configuration, we brought the spatial resolution near the half-wavelength diffraction limit, which for long had been thought to be inaccessible in Brillouin microscopy due to the spectral broadening^22^. Recent progress on stimulated Brillouin scattering has shown the potential to significantly decrease the data acquisition time^29^,^30^, which may enable real-time mechanical imaging of cell dynamics.

In this work, Brillouin microscopy was validated in a controlled setting to investigate the subcellular biomechanical properties in healthy primary cells *in vitro.* The data presented here indicate that separate cellular compartments such as the cytoplasm, nuclear membrane, and nucleoli have markedly different mechanical properties. In addition, cytoplasmic stiffness was significantly reduced after administration of the drug latrunculin-A. In contrast, nucleoli did not exhibit significant changes in stiffness in response to latrunculin-A. These observations are consistent with our hypotheses because latrunculin-A acts by preventing polymerisation of the actin cytoskeleton, a protein that is omnipresent in the cytoplasm but is almost absent from the nucleus and nucleoli. As such, these results validate Brillouin microscopy as a technique to investigate the cellular and subcellular mechanical properties of a volume of cells *in vitro*, and their changes over time or in response to external stimuli. In addition to the pharmacological stimulus in the form of latrunculin-A that was used in the current study, Brillouin microscopy could also be used to study the effect of other stimuli such as mechanical or electrical stimuli on subcellular mechanics.

In Brillouin images, the cytoplasm does not exhibit the fibre-like appearance that is typical for the cytoskeleton because the diameter of individual cytoskeletal fibres (typically ~7 nm for F-actin filaments and ~25 nm for microtubules^31^,^32^) is below the spatial resolution of the system. Moreover, any structures in the ~5-300 nm size range would result in Lamb modes rather than a bulk longitudinal peak, which standard VIPA spectrometers are not able to resolve due to saturation of the Rayleigh lines and lack of spectral resolution. However, the Brillouin microscope is able to register changes in cytoplasmic stiffness due to cytoskeletal changes, as is evidenced by the reduced cytoplasmic stiffness after exposing the cells to latrunculin-A.

Subcellular biomechanics play an important role in cancer, where cell motility is an important determinant in the metastasis of the disease, a deeper understanding of nuclear mechanics in healthy and diseased cells could lead to the identification of new targets for cancer therapies. Altered nuclear mechanics are also likely involved in the diseases Emery-Dreifuss muscular dystrophy and Hutchinson-Gilford progeria. Both diseases are associated with mutations in the *LMNA* gene that encodes the lamin A and C proteins that form part of the nuclear envelope. Lammerding *et al.*, showed that lamin A/C deficient cells have reduced nuclear stiffness, increased nuclear fragility, experience increased cell death under mechanical strain, and exhibit altered mechanotransduction^33^,^34^. As such, non-invasive Brillouin microscopy can help elicit changes in subcellular mechanics and its contribution to processes such as cancer metastasis and diseases such as muscular dystrophy and progeria.

The results presented here establish Brillouin microscopy as a versatile and broadly applicable technique to investigate subcellular biomechanics, and to study how external stimuli lead to changes in the mechanical properties of cells. The high spatial resolution, three dimensional imaging capabilities, and non-invasive nature of the technique, create new opportunities to further the field of cellular mechanics and its involvement in the pathophysiology of diseases such as cancer, atherosclerosis, glaucoma, progeria and muscular dystrophy.

## Methods

### Brillouin scattering

Spontaneous Brillouin scattering arises from the interaction of light with local spontaneous acoustic waves propagating in a material at the hypersound velocity *V*. The Brillouin spectrum is composed of a Stokes and an Anti-Stokes peak typically shifted by 1–20 GHz from the central elastic (Rayleigh) peak. The frequency shift associated with the Brillouin scattered light is given by ν_B_ = (2*n*/λ)*V*sin(θ/2), where *n* is the refractive index, λ is the incident wavelength and θ is the scattering angle. The real part of the longitudinal modulus is associated with the hypersound velocity through the relation M′ = *ρV*^*2*^, where *ρ* is the material density.

### Confocal Brillouin Microscope

A custom confocal Brillouin microscope was built to yield three-dimensional biomechanical images with a subcellular spatial resolution. A CW single longitudinal mode laser (λ = 561 nm, Cobolt Jive) was used as a light source in all experiments. The beam was first cleaned from optical aberrations by a single mode optical fibre, and then magnified by a 10x beam expander. To obtain a submicron spatial resolution, the beam was focused to the sample by a high NA oil-immersion microscope objective (100x, NA = 1.3, Olympus UPLNFN 100XOI). The system point spread function (PSF) associated with the instrumental spatial resolution was characterised through an image stack of a sub-diffraction sized microsphere (Supplementary Figure S3). The samples were placed on a stabilised motorised stage (Prior Scan III) mounted on an inverted microscope (Olympus IX71) to perform fast three-dimensional scanning. The optical power at the object plane was maintained below 5 mW to avoid sample damages and heating. The light scattered by the sample was coupled into a single mode fibre, which enabled strict confocality and flexible beam delivery to a customised two-stage VIPA spectrometer (Supplementary Figure S4)^35^. This had a spectral contrast of approximately 60 dB (Supplementary Figure S5), and a spectral resolution of ~350 MHz (Supplementary Figure S6). A high spectral contrast is indeed required to measure the Brillouin peaks when strong elastic scattering and specular reflections arise from the sample^36^. Each Brillouin spectrum was acquired by an sCMOS camera (Andor Neo 5.5) with a data acquisition time of 0.2 sec. A LabView software was developed to drive both the stage and the camera simultaneously. A Brillouin spectrum of water (Supplementary Figure S6) was acquired as a spectral reference before the scanning process. Three-dimensional spatial distributions of the longitudinal modulus across cells were yielded after optical sectioning and computational processing of the Brillouin spectra acquired.

### Data processing

Raw Brillouin spectra were computationally processed using custom MATLAB codes. Least square Lorentzian fittings were performed to localise the Brillouin peaks along the dispersion axis (Supplementary Figure S7), where each Brillouin peak was composed of more than 10 data points as in standard Brillouin spectroscopy schemes^37^. The well-known spectral shift of water (ν_B_ ≈7.4 GHz for θ = 180°) was used as a reference for the spectral analysis. Conversion of the Brillouin frequency shift to the elastic longitudinal modulus was performed through the relationship M′ = *ρ*(λν_B_/2*n*)^2^, where we assumed a cellular density of ρ = 1080 Kg/m^3^ and refractive index of *n* = 1.38^38^.

### Cell culture

No. 1.5 glass coverslips were prepared for cell seeding by cross-linking a 100 μm thin layer of polyacrylamide onto the glass following a protocol by Tse and Engler^39^. The polyacrylamide gels were then incubated with 1% gelatine solution to promote cell attachment. Porcine aortic endothelial cells from primary isolations were seeded onto the specially prepared coverslips and maintained in phenol-free DMEM (Sigma Aldrich D5921) supplemented with 10% serum, glutamine, penicillin and streptomycin at 37 °C and 5%CO_2_. After four days, cells were transferred to a specialist life cell imaging solution (Gibco A14291DJ) to maintain stable pH during Brillouin imaging. First, a baseline-image of the cells was acquired through a series of z-stacks. For the latrunculin-A experiment, the drug (Sigma Aldrich A5163) was added to the imaging solution at a final concentration of 500 nM.

### Statistical analysis

Normality of the Brillouin measurements was confirmed using a Kolmogorov-Smirnov test. The statistical difference between longitudinal moduli of different organelles/stimuli in different cells was investigated using an analysis of covariance (ANCOVA) test. The Brillouin measurements were considered as a continuous independent variable, and the organelle/stimulus, and the cell number as nominal covariates. The linear regression model was adjusted based the data using a stepwise regression approach. All statistical analyses were performed using MATLAB version 2014a (The Mathworks, Natick, MA, USA).

## Additional Information

**How to cite this article**: Antonacci, G. and Braakman, S. Biomechanics of subcellular structures by non-invasive Brillouin microscopy. *Sci. Rep.* **6**, 37217; doi: 10.1038/srep37217 (2016).

**Publisher’s note:** Springer Nature remains neutral with regard to jurisdictional claims in published maps and institutional affiliations.

## Supplementary Material

Supplementary Information
