# Corrigendum: Biomechanics of subcellular structures by non-invasive Brillouin microscopy

**DOI:** 10.1038/srep46789

**Published:** 2017-05-09

**Authors:** Giuseppe Antonacci, Sietse Braakman

Scientific Reports 6: Article number: 3721710.1038/srep37217; published online: 11 15 2016; updated: 05 09 2017

The authors wish to acknowledge other contributors and other sources of funding.

The Author Contributions statement should read:

G.A. and S.B. contributed to the design of the experiments. G.A. contributed to the development of the microscope. G.A. and S.B. collected the data. S.B. contributed to the development of cell protocols and sample preparation. G.A. and S.B. wrote the manuscript.

The Acknowledgements should read:

The authors would like to thank Prof Peter Török and Dr Carl Paterson for their advice, guidance on and contributions to the design of the microscope; Prof Török and Dr Darryl Overby for their advice and guidance on the experimental design; and Dr Overby for his advice and guidance on the development of cell protocols and sample preparation. Mr Martin Kehoe and Mr Simon Johnson are acknowledged for their valuable help in designing and manufacturing parts of the optomechanical setup. The authors wish to thank Margherita Rossi for her contribution to the design of the figures. The authors acknowledge funding from the EPSRC Doctoral Training Account (G.A.) and a PhD studentship from the Department of Bioengineering, Imperial College London (S.B.), as well as funding from a National Institutes of Health grant EY019696 (Dr Overby) and EPSRC Pathways to Impact Grant (Prof. Török).

