# Supplementary material for: Biomechanics of subcellular structures by non-invasive Brillouin microscopy
Source: Sci Rep. 2016 Nov 15;6:37217. doi: 10.1038/srep37217 (PMC5109042; doi:10.1038/srep37217)
Supplement: Supplementary Information [file srep37217-s1.pdf]

Biomechanics of subcellular structures by non-invasive Brillouin microscopy

Giuseppe Antonacci[1,2*], Sietse Braakman[3]

Supplementary Figures

Figure S1 – Box and whisker plot for different subcellular compartments

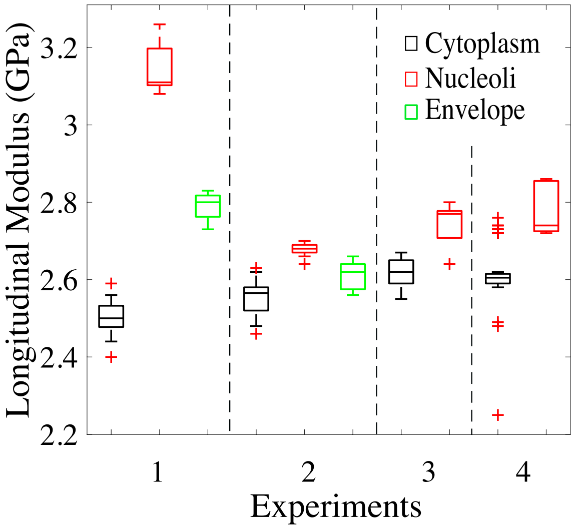

Figure S1 - A box and whisker plot representing the difference in longitudinal modulus between different subcellular compartments in four separate experiments. The whiskers represent the 95% confidence interval for each set of measurements and do not overlap between the nucleolar and cytoplasmic measurements within each individual cell. When considering all cells, nucleolar longitudinal modulus was found to be significantly higher than cytoplasmic longitudinal modulus ($p<0.0001$, ANCOVA analysis).

Figure S2 – Box and whisker plot for cellular biomechanics in response to latrunculin-A

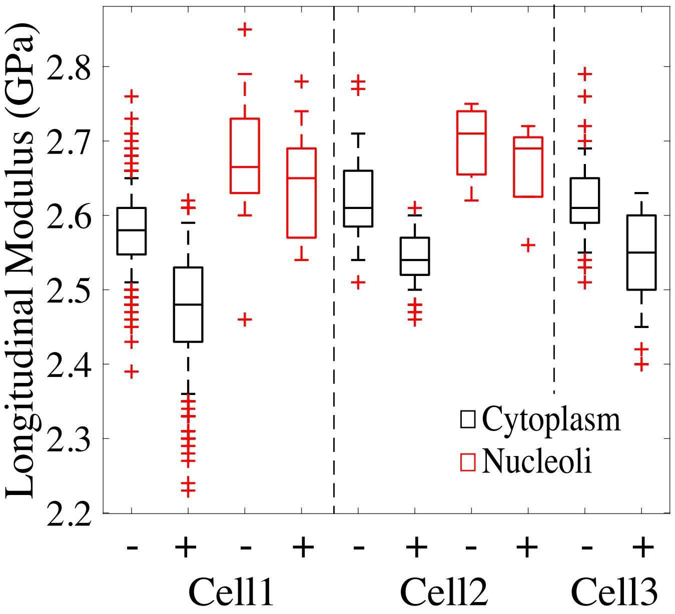

Figure S2 - A box and whisker plot representing the difference in longitudinal modulus of the cytoplasm and the nucleoli in three experiments before (-) and after (+) the administration of latrunculin-A. The whiskers represent the 95% confidence interval for each set of measurements and do not overlap between the nucleolar and cytoplasmic measurements within each individual cell. When considering all cells, cytoplasmic longitudinal modulus was found to decrease significantly in response to latrunculin-A ($p<0.0001$, ANCOVA analysis, whereas the nucleolar modulus showed no significant change ($p=0.65$).

Figure S3 – PSF characterisation

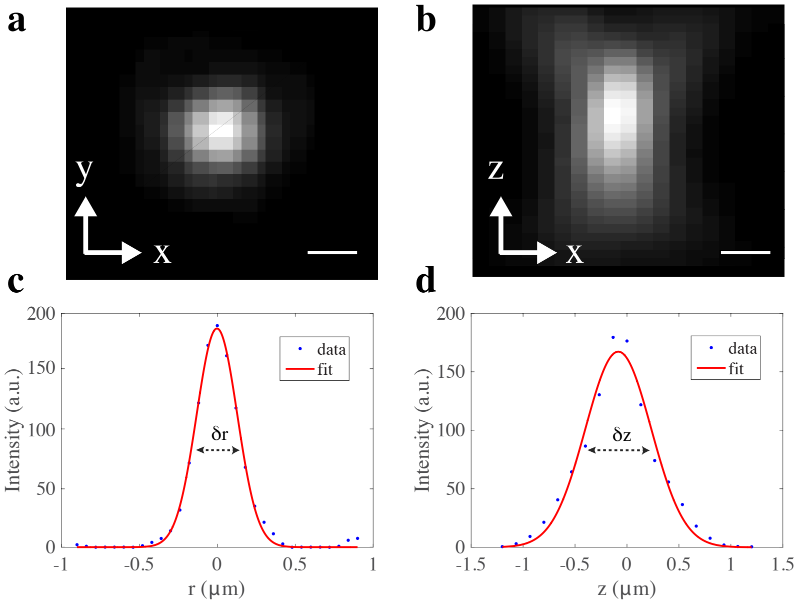

Figure S3 – PSF calibration measurements. A sub-diffraction sized polystyrene microsphere of 200 nm diameter (ThermoFischer) was imaged with the confocal microscope through a stack of frames in both lateral (a) and axial (b) planes. Scale bar, 200 nm. The system PSF was reconstructed from the intensity profile of the microsphere along the transverse (c) and the longitudinal (d) direction. A Gaussian fit was performed to measure the FWHM associated with the lateral and the axial spatial resolution of the microscope, which were found to be δr=(304±23) nm and δz=(693±32) nm respectively. These findings are in good agreement with the theoretical predictions based on the conventional Rayleigh criterion for confocal microscopes.

Figure S4 – Two-stage VIPA spectrometer

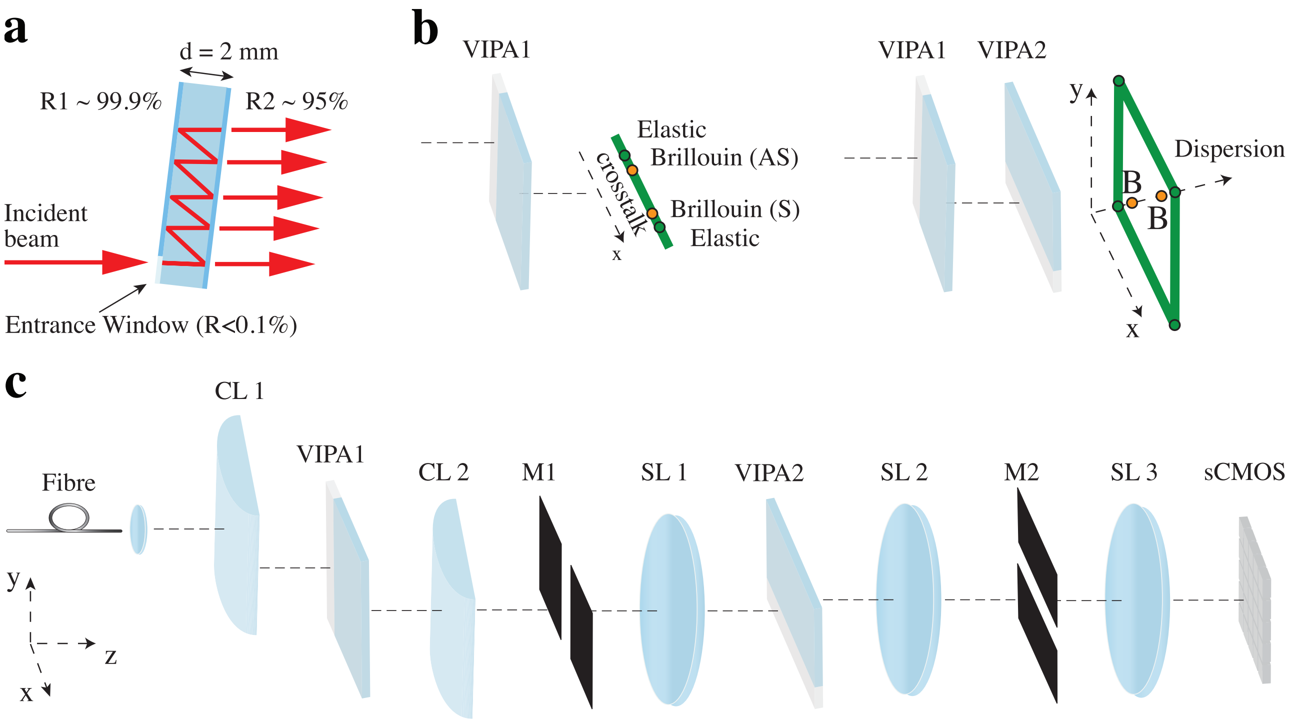

Figure S4 – Diagram of a virtually imaged phase array (VIPA) (**a**). Light is coupled through an anti-reflection coated (R<0.1%) entrance window to minimise insertion losses. The input beam experience multiple internal reflections and the transmitted interference fringes give rise to the spectrum of light as in a standard Fraby-Pérot etalon. When two crossed VIPA etalons of equal power are distributed in tandem, the spectral dispersion axis rotates by 45° to the crosstalk lines (green lines) resulting in a higher extinction (**b**). This principle is at the base of the operation of a two-stage VIPA spectrometer (**c**). The scattered light delivered by a single mode fibre is focused by a cylindrical lens (CL1) to the first VIPA entrance window. A relay telescope formed by a cylindrical lens (CL2) and a spherical lens (SL1) enables the insertion of a spatial mask (M1) to cut the crosstalk lines along the y-axis. A second mask (M2) equally removes the crosstalk along the x-axis so as to filter the Brillouin signal with a total throughput efficiency of ~30%. The resulting spectral pattern is acquired by an sCMOS camera.

Figure S5 – Spectral extinction

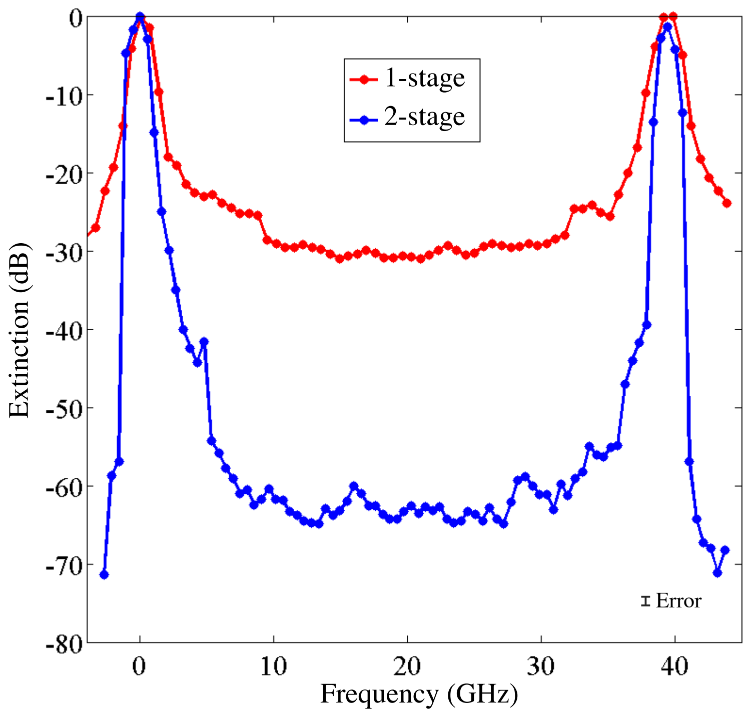

Figure S5 – Spectral extinction of a single- and a two-stage VIPA spectrometer. To overcome the limited dynamic range of the CCD camera, the spectral profile was reconstructed after scaling the data according to a set of calibrated neutral density filters used to attenuate the laser intensity level. A two-stage VIPA spectrometer gives a spectral extinction of approximately 60 dB, i.e. ~30 dB more than the extinction given by a single-stage VIPA spectrometer.

Figure S6 – Spectral resolution

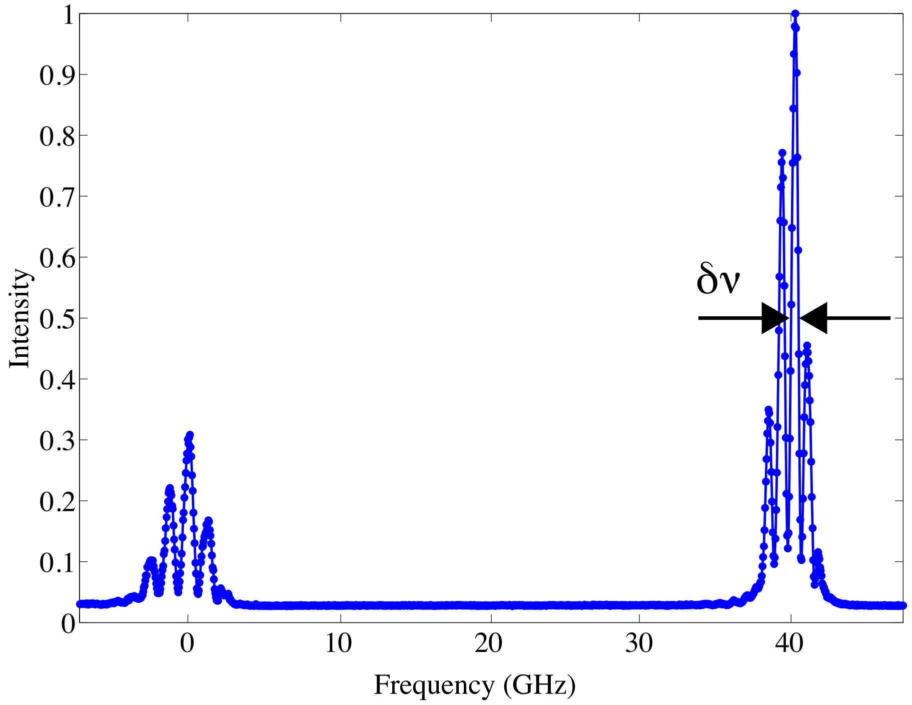

Figure S6 – Spectrum of a non-monochromatic HeNe laser. The narrow spectral lines associated with the 4 longitudinal frequency modes of the laser (Uniphase, cavity length ~420 mm) are shown along two consecutive interference orders (i.e. a single FSR) of the VIPA spectrometer. Individual laser modes were resolved by the VIPA spectrometer and from the measure of the peak FWHM we obtained a spectral resolution of $\delta\nu$=(354±20) MHz.

Figure S7 – Brillouin spectrum of water

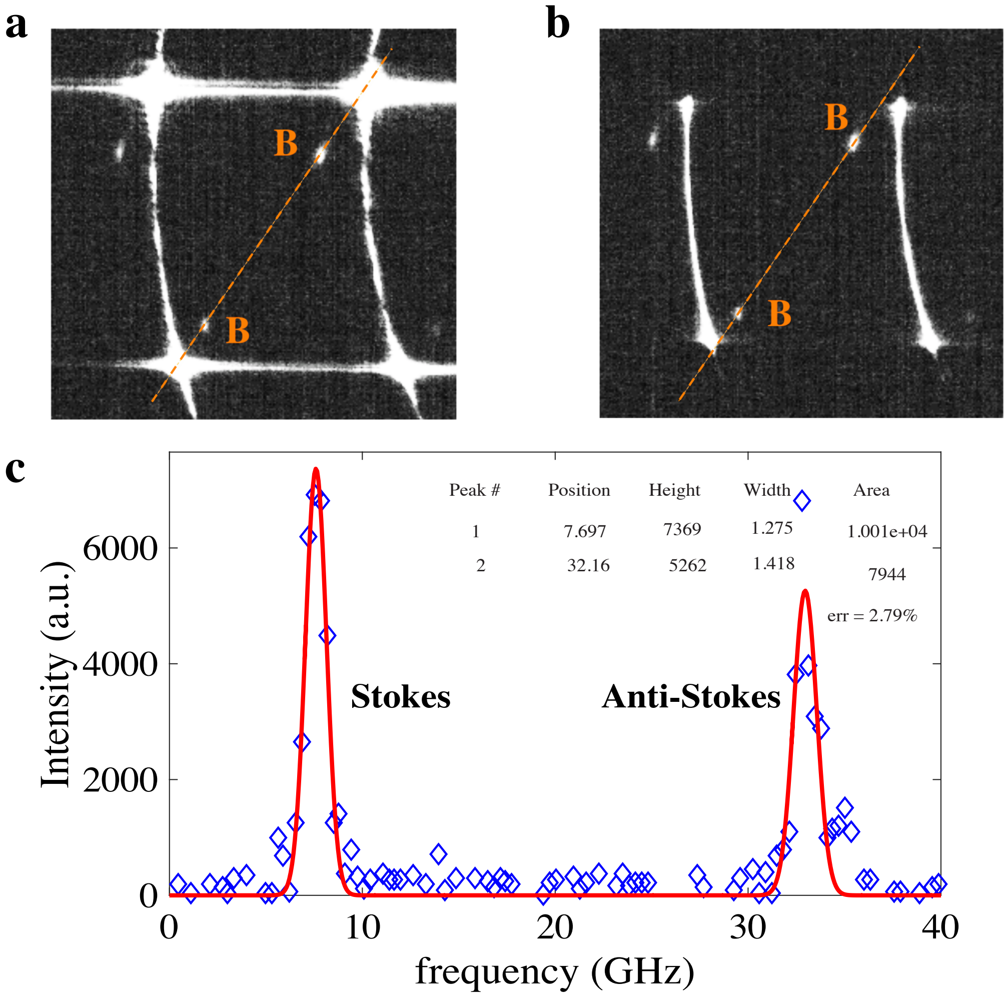

Figure S7 – Brillouin spectrum of water acquired by a two-stage VIPA spectrometer (**a**). The crosstalk signal due to collection of high specular reflections and elastically scattered light can easily overcome the weak Brillouin peaks (B). The application of a spatial mask removes the elastic crosstalk lines along one direction (**b**). A Lorentzian fit (red line) is performed along the dispersion axis (dashed line in **b**) to measure the spectral shift of the Brillouin peaks (**c**).
